# Commentary on “Incidence and relative risk of colorectal cancer in autoimmune diseases: a global pooled-analysis with more than 91 million participants”

**DOI:** 10.1097/JS9.0000000000002986

**Published:** 2025-07-08

**Authors:** Runbing Xu, Haomin Zhang, Bao Sun

**Affiliations:** a Department of Hematology and Oncology, Beijing University of Chinese Medicine Affiliated Dongzhimen Hospital, Beijing, China; b Department of Dermatology, Beijing University of Chinese Medicine Affiliated Dongzhimen Hospital, Beijing, China; c Department of Pharmacy, The Second Affiliated Hospital of Xi’an Medical University, Xi’an, Shaanxi, China

*Dear Editor,*

We read with great interest the timely and comprehensive meta-analysis by Cheng and colleagues, “Incidence and relative risk of colorectal cancer in autoimmune diseases: a global pooled-analysis with more than 91 million participants”[1]. This extensive global pooled analysis offers important insights, affirming a generally elevated incidence and relative risk of colorectal cancer (CRC) among patients with autoimmune diseases, across sub-localizations, sexes, and geographical regions. However, the observed protective effect of rheumatoid arthritis (RA) against CRC challenges the conventional view that chronic inflammation universally promotes carcinogenesis, which warrants deeper exploration. This correspondence is compliant with the TITAN Guidelines 2025 – governing declaration and use of AI[2].

First, recent studies suggest this protection is driven by immune-mediated inflammation. Specifically, high expression of RA-related genes, particularly *IL6R*, shows a negative correlation with colon adenocarcinoma, suggesting that certain patterns of immune activation may fortify anti-tumor surveillance or establish a microenvironment that is unfavorable for CRC development[3]. Dissecting the distinct immune pathways underlying RA may therefore hold the key to understanding this protective phenomenon.

Secondly, pharmacotherapy introduces further complexity. Nonsteroidal anti-inflammatory drugs, frequently used in RA, are known to reduce CRC risk. Methotrexate, a cornerstone of RA treatment, has been linked to decreased CRC risk in women in a dose-dependent manner[4]. While concerns about biologic agents (TNF-α inhibitors) and malignancy risk have been raised, data from large-scale reviews have not demonstrated a significant increase in CRC incidence[5]. In contrast, the use of corticosteroids may be deleterious in oncologic settings, potentially impairing survival and promoting tumor progression[6]. Robust pharmaco-epidemiological studies are required to disentangle the influence of underlying disease biology from that of therapeutic exposures, accounting for cumulative dosing and drug interactions.

Thirdly, another emerging dimension is the gut microbiome. RA is characterized by distinct dysbiosis, including alterations in *Proteobacteria, Firmicutes*, and genera such as *Klebsiella* and *Prevotella*[7]. Given the pivotal role of gut microbial composition in both RA pathogenesis and CRC development, it is plausible that these shifts in microbial ecology modulate colorectal carcinogenesis. Functional profiling of these microbial communities could uncover novel chemopreventive targets.

In conclusion, the inverse relationship between RA and CRC reflects a complex interplay between immune dysregulation, pharmacologic interventions, and host–microbe interactions. A deeper mechanistic understanding is essential to inform personalized CRC risk assessment and targeted surveillance strategies for individuals with autoimmune diseases.

## Data Availability

All data are included in the article.
